# Tunable Chemokine Production by Antigen Presenting Dendritic Cells in Response to Changes in Regulatory T Cell Frequency in Mouse Reactive Lymph Nodes

**DOI:** 10.1371/journal.pone.0007696

**Published:** 2009-11-06

**Authors:** Valentina Dal Secco, Cristiana Soldani, Claire Debrat, François Asperti-Boursin, Emmanuel Donnadieu, Antonella Viola, Adelaida Sarukhan

**Affiliations:** 1 Istituto Clinico Humanitas I.R.C.C.S., Milan, Italy; 2 Institut Cochin, Université Paris Descartes, CNRS 8104, INSERM U567, Paris, France; 3 Department of Translational Medicine, University of Milan, Italy; University of Alabama-Birmingham, United States of America

## Abstract

**Background:**

Although evidence exists that regulatory T cells (Tregs) can suppress the effector phase of immune responses, it is clear that their major role is in suppressing T cell priming in secondary lymphoid organs. Recent experiments using two photon laser microscopy indicate that dendritic cells (DCs) are central to Treg cell function and that the *in vivo* mechanisms of T cell regulation are more complex than those described *in vitro*.

**Principal Findings:**

Here we have sought to determine whether and how modulation of Treg numbers modifies the lymph node (LN) microenvironment. We found that pro-inflammatory chemokines—CCL2 (MCP-1) and CCL3 (MIP-la)—are secreted in the LN early (24 h) after T cell activation, that this secretion is dependent on antigen-specific DC–T cell interactions, and that it was inversely related to the frequency of Tregs specific for the same antigen. Furthermore, we demonstrate that Tregs modify the chemoattractant properties of antigen-presenting DCs, which, as the frequency of Tregs increases, fail to produce CCL2 and CCL3 and to attract antigen-specific T cells.

**Conclusions:**

These results substantiate a major role of Tregs in LN patterning during antigen-specific immune responses.

## Introduction

The immune system has developed several sophisticated regulatory mechanisms that ensure tolerance towards self-antigens and moderate inflammation induced by pathogens and environmental insults. Among these mechanisms, suppression of T cell functions by regulatory T cells (Treg) is crucial and Tregs are now considered as the primary mediators of peripheral tolerance. Naturally-occurring Tregs are positively selected in the thymus, presumably by self-antigens that remain to be defined, and express the FoxP3 transcription factor, which is essential for their regulatory function [1], [2] but not for their lineage determination [3]. The role of Treg cells in maintaining tolerance to self-antigens is evidenced in mice and individuals that lack FoxP3 and that develop a profound autoimmune-like lymphoproliferative disease [4], [5]. However, Treg cells may also block beneficial responses, as reported for antitumor immunity [6], and interfere with the complete removal of pathogens [7]. Thus, it is of obvious importance to define the mechanisms of *in vivo* T cell regulation, not only to better understand the process of peripheral tolerance but also to develop effective approaches for the clinical manipulation of Treg cells.

Although evidence exists that Tregs can suppress the effector phase of immune responses, it is clear that their major role is in suppressing T cell priming in secondary lymphoid organs. A considerable number of experiments performed *in vitro* have shown that Treg cells depend on direct cell-cell contact to mediate their inhibitory activity, and have suggested that the major mechanisms described (inhibitory cytokines, cytolysis and metabolic disruption) act directly on the effector T cell (reviewed in[8]). However, recent intravital microscopy experiments have demonstrated that the presence of Tregs in the lymph node (LN) decreases the frequency of stable contacts between self-reactive T cells and dendritic cells (DCs) that supposedly present the cognate antigen [9], [10]. Furthermore, no detectable direct interaction between suppressed T cells and Tregs was observed during the *in vivo* experiments; in contrast, direct interaction between antigen-bearing DCs and Tregs was reported, suggesting that the mechanisms of *in vivo* regulation are much more complex than those described *in vitro* and likely involve DCs.

Chemokines control homeostatic circulation of leukocytes as well as their movement to sites of inflammation or injury. For example, CCR7 and its ligands, CCL19 and CCL21, direct the trafficking of T cells, B cells [11], [12] and activated DCs [13], [14], to and within lymph nodes. CCR5 and its ligands facilitate efficient CD8 T cell priming within the LN [15]. CCL2, through its receptor CCR2, can recruit monocytes, immature DC and natural killer (NK) cells under inflammatory conditions [16], [17], [18], [19], [20].

Interestingly, it has been recently reported that ablation of Tregs unexpectedly increases susceptibility to virus infection, as a consequence of enhanced production of proinflammatory cytokines and chemokines in the LN, paralleled by a reduced or delayed recruitment of inflammatory DCs, NK and T cells to the sites of infection [21]. It is thus conceivable that, *in vivo*, Tregs modify the local lymphoid microenvironment and, consequently, DC behavior or functions. To address this question in a setting that would allow us to know what Tregs are responding to, we used a TCR transgenic mouse model in which regulatory and conventional T cells with the same antigen specificity develop. Furthermore, this mouse model allows studying the effect of tunable fluctuations in Treg number on the inflammatory LN environment, a condition that may resemble what observed in some pathological conditions (reviewed in [22]).

## Materials and Methods

### Ethics Statement

Procedures involving animals and their care conformed with institutional guidelines (authorisation n. 11/2006-A from the Italian Ministry of Health) in compliance with national (4D.L. N.116, G.U., suppl. 40, 18-2-1992) and international law and policies (EEC Council Directive 86/609, OJ L 358,1,12-12-1987; NIH Guide for the Care and Use of Laboratory Animals, US National Research Council 1996). All efforts were made to minimize the number of animals used and their suffering.

### Mice

BALB/c (H-2d) mice were from Charles River Laboratories (Italy). TCR-HA transgenic mice expressing a TCRαβ specific for peptide 111–119 from influenza virus hemagglutinin (HA) presented by I-E^d^ have been previously described [23], are on the BALB/c background and are referred to as single transgenic (stg). These mice were crossed with mice expressing influenza HA under the control of the ubiquitous *pgk* promoter to generate TCR-HA x pgk-HA double-transgenic mice [24], referred to as dtg. All mice were used between 5 and 8 weeks of age.

### Antibodies and Reagents

The clonotypic 6.5 mAb, which recognizes the transgenic TCR-HA, was produced in our laboratory and was used coupled to biotin or PE. All other antibodies for flow cytometry were purchased from BD Pharmingen. Cells were analyzed on a flow cytometer (FACS Canto; Becton Dickinson). Cell sorting was peformed using a FACS Aria (Becton Dickinson). Facs data were analysed using Diva software and FlowJo software. LPS (Escherichia coli 026:B6) was purchased from Sigma-Aldrich. The HA peptide (SVSSFERFEIFPK) was purchased from Invitrogen.

### Cell Isolation and Preparation

For *in vitro* experiments, conventional (CD4^+^CD25^−^6.5^+^) or regulatory (CD4^+^CD25^+^6.5^+^) T cells specific for HA were stained with anti-CD4, anti-CD25 and 6.5 antibodies and sorted on a Facs Aria (BD Biosciences).

For adoptive transfer experiments, T cells were obtained from the LNs of stg or dtg mice, incubated with the biotinylated 6.5 mAb, and positively selected with anti-biotin MACS microbeads (Miltenyi Biotec). Purity was always >85%.

Dendritic cells (DC) were obtained from the bone marrow of BALB/c mice and were grown for 10 days in complete IMDM supplemented with 30% supernatant of GM-CSF expressing fibroblasts. In some cases, DCs were labelled with 7 µM 5- and 6-(4-chloromethyl) benzoylamino-tetramethylrhodamine (CMTMR) (Invitrogen) and pulsed with HA peptide (5 µg/ml) for one hour, washed extensively in PBS and injected s.c. in the hind footpads.

### In Vitro Proliferation and Regulation Assays

All assays were performed in complete IMDM (Gibco), supplemented with 2-mercaptoethanol (Gibco) and 10% FCS. FACS-sorted CD4^+^6.5^+^CD25^−^ or CD25^+^ T cells (2.5×10^4^) obtained from stg or dtg mice were incubated with DCs (1.5×10^4^) in flat bottom 96 well plates. In some wells, FACS-sorted CD4^+^6.5^+^CD25^−^ or CD25^+^ T cells obtained from dtg mice were added to CD4^+^6.5^+^CD25^−^ from stg mice at a ratio of 1∶1. After 3 days of culture, supernatants were collected for the quantification of cytokines and 1 µCi ^3^H-methyl-thymidine was added for an additional 16 h. All conditions were performed in triplicates.

### Immunization and Adoptive Transfers

Stg or dtg mice were immunized with 0.5–1×10^6^ CMTMR labelled DCs, loaded or not with peptide, or with soluble HA peptide with LPS (1 µg HA peptide and 0.5–1 µg LPS per footpad) by s.c. injection into the footpad. In adoptive transfer experiments, CFSE-labelled 6.5^+^ T cells from stg mice (1×10^6^ cells) were adoptively transferred by i.v. injection into BALB/c recipient mice that had received or not 2×10^6^ 6.5^+^ T cells from dtg mice 6–18 h earlier. Recipients were immunized as described above. Immunized mice were sacrificed at different time points and the popliteal draining LNs (dLNs) were collected and treated with 1.6 mg/ml collagenase IV (Sigma-Aldrich) and 0.2 mg/ml DNAse (Roche) at 37°C for 30 min. Cells were washed in PBS, counted and stained with specific antibodies. Contralateral popliteal or axillary lymph nodes were used as control non-draining LN.

### Cytokine and Chemokine Detection

Cytokine and chemokine concentrations were quantified from supernatants of *in vitro* cultures or from homogenized LNs from immunized mice, using the ELISA duoset kits (R&D Systems) according to the manufacturer's instructions.

### Confocal Microscopy

BALB/c mice were adoptively transferred with CFSE-labelled 6.5^+^ T cells from stg or dtg mice and one day later immunized with HA-loaded CMTMR+ DCs in the hind footpad. After 24 hours, dLNs were recovered and immediately frozen in OCT. 10 µm cryostat sections were cut, fixed in formalin 4% for 10 min and rehydrated in PBS. The nuclei were counterstained with Hoechst (1 µg/ml, Invitrogen) and the slides were mounted with ProLong (Invitrogen). Acquisition of images was made by confocal microscopy Fluoview FV1000 (Olympus, Tokio, Japan) and an oil immersion objective (60×1.4 NA Plan-Apochromat; Olympus). To perform DC and T counts, random-picked 250×250 µm quadrants at 40× magnification containing at least one DC were considered and the T/DC ratio was calculated.

For CCL2 and CCL3 stainings, fixed 10 µm cryostat sections were incubated with the primary biotinylated CCL2 or CCL3 (MIP-1α) antibodies (R&D Systems) and revealed with Alexa647-streptavidin or Alexa647-anti-goat antibody respectively (Invitrogen). Negative controls included sections incubated with the secondary antibodies alone.

To perform colocalization analysis, the images were obtained with a 60×1.4 NA objective with a resolution of 800×800 and a laser excitation at 405, 488, 543 and 633 nm. Differential interference contrast (Nomarski technique) was also used. The extent of co-localization of two given labels was measured using the ‘Co-localization’ module of Imaris 5.0.1, 64-bit version (Bitplane AG, Saint Paul, MN). For each data set, 10 individual cells were analyzed for co-localization.

To quantify the labelling for CCL2 and CCL3, a ROI on CMTMR^+^ DCs was drawn and the parameter ‘Percentage of material co-localized’, which includes both the number of voxels and their intensities, was calculated.

### Statistical Analysis

Results are expressed as means ± standard deviation. Groups were compared by using non-paired Student *t* test and the nonparametric Mann-Whitney test. p values <0.05 (*), 0.01 (**) and 0.001 (***) were considered significant. For the analysis of T:DC co-localization a non-parametric ANOVA test was performed and the p value for the difference between the upper two quartiles and the lower two quartiles of each sample was determined by Dunn's multiple comparison test. Statistical analysis was performed using the Prism software (graphPad).

## Results

### TCR Transgenic Mice with Different Tconv:Treg Cell Ratios

**Figure 1:**
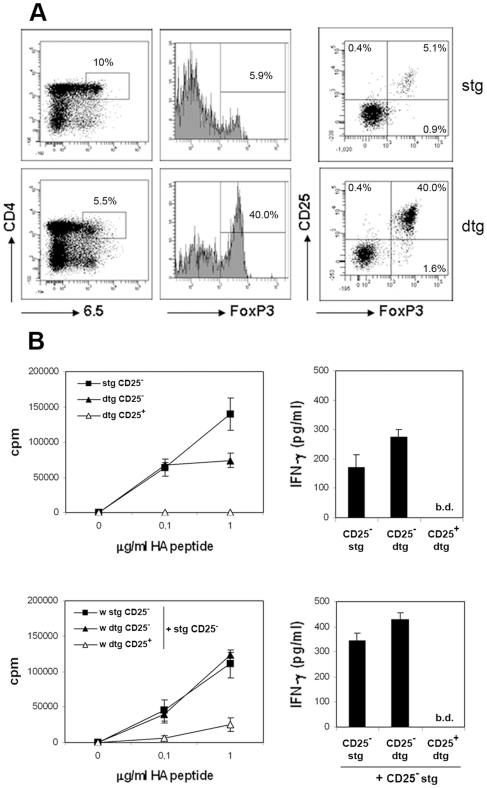
Tconv:Treg ratio and their proliferative capacity in stg and dtg mice. (A) Cell suspensions obtained from the LNs of stg or dtg mice were stained for CD4, 6.5, CD25 and FoxP3 and analysed by flow cytometry. Living cells were gated for CD4 and 6.5 expression. One representative dot plot is shown. (B) LN cells from stg or dtg mice were stained and sorted by flow cytometry according to CD4, CD25 and 6.5 expression. 2.5×10^4^ sorted T cells were cultured in triplicate wells with 1.5×10^4^ DCs and different doses of HA peptide. After 70 h of coculture, supernatants were collected for IFNγ measurement and thymidine was added for an additional 16 h in order to measure proliferation (upper panels). In parallel, the ability of CD25^−^ vs CD25^+^ dtg sorted cells to inhibit proliferation and IFNγ production of naive HA-specific T cells (stg CD25−) was analyzed (lower panels). Shown is one out of three independent experiments that gave similar results. b.d. = below detection.

Mice expressing a transgenic TCR that is specific for a peptide from the influenza virus hemagglutinin (HA), restricted to MHC I-E^d^ molecules and recognized by the clonotypic antibody 6.5, have been previously described [23], [25]. In these mice, the frequency of FoxP3^+^ cells is low when gating among CD4^+^6.5^+^ cells (6.2+0.9%). When these TCR-HA single transgenic mice are crossed to mice expressing HA under the ubiquitous pgk promoter (TCR-HA x pgk-HA double transgenic mice), a high proportion of CD4^+^ T cells expressing the transgenic TCR in periphery are CD25^+^ and are capable of regulating HA-specific responses *in vitro* and *in vivo* [24]. In agreement, the frequency of FoxP3^+^ cells among the CD4^+^6.5^+^ population is 8–10 times higher in double transgenic (dtg) than in single transgenic (stg) mice (Fig. 1A). CD4^+^6.5^+^CD25^−^ cells from dtg mice, which represent the majority of the FoxP3^−^ population, can be considered as T conventional (Tconv) cells, because they are capable of proliferating and secreting IFNγ in response to various peptide doses and, in contrast to their CD25^+^ counterparts, do not suppress proliferation of 6.5^+^CD25^−^ cells from stg mice (Fig. 1B).

We therefore used these mice (TCR-HA stg and TCR-HA x pgk-HA dtg) to evaluate the effects of a ten-fold difference in the Tconv:Treg cell ratio (15.2±2.4∶1 in stg mice vs. 1.1±0.2∶1 in dtg mice) on the LN microenvironment and DC behavior.

### Decreased Numbers of DCs in the Draining LNs Is Associated to a High Frequency of Treg Cells

In order to address DC recruitment and retention within LNs containing low or high frequencies of antigen-specific Treg cells, 1×10^6^ CMTMR-labelled, bone-marrow derived DCs that were loaded or not with HA peptide were injected in the footpad of stg or dtg mice. At various times after injection, draining popliteal LNs (dLNs) were recovered and cells were analyzed by flow cytometry.

**Figure 2:**
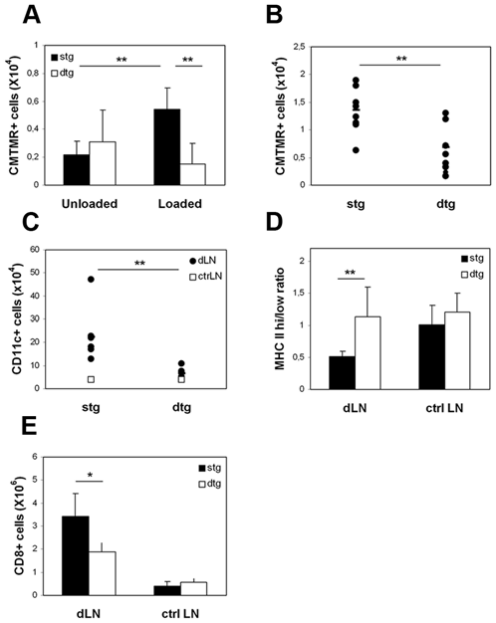
Effects of Tregs on DC migration towards dLNs. DCs were stained with CMTMR, loaded or not with HA peptide and injected into the footpads of stg or dtg mice. dLNs were harvested at day 1 (A) or day 2 (B–E) post-injection, collagenase-digested, and analysed by flow cytometry to determine absolute numbers of CMTMR^+^, transferred (A, B) and CMTMR^−^ CD11c^+^, endogenous (C) DCs, the ratio of MHC class II high/low CD11c^+^ cells (D) and the absolute numbers of CD8^+^ cells (E). The figures shown correspond to pooled data from three independent experiments; in each experiment two to four mice per group were used.

In accordance with early studies [26], we found that in stg mice antigen-loaded DCs migrate and accumulate in dLNs to a greater extent than unloaded DCs (Fig. 2A). The absolute number of antigen-loaded DCs recovered at day 1 from the dLNs of dtg mice was significantly lower compared to that recovered from the stg ones. This difference was also observed after two days (Fig. 2B) and tended to disappear by day 4 (not shown). TCR-HA transgenic mice contain some CD8^+^ cells that express the transgenic TCR and could be contributing to DC cytotoxicity. To exclude this possibility, we performed annexin V staining on dLN suspensions as well as TUNEL, caspase 3 and caspase 9 immunofluorescence on dLN tissue sections from stg and dtg mice that received CMTMR-labelled DCs loaded with HA peptide. We could not detect any significant apoptosis among CMTMR-positive DCs injected into either recipient (Fig. S1) indicating that the decreased number of HA-loaded DCs in the dLN of dtg mice at days 1 and 2 was not due to increased DC apoptosis within the draining lymph node.

Since at 48 h the total cellularity of the dLNs was increased in the stg mice as compared to the dtg mice (5.4×10^6^±2.0 vs. 2.5×10^6^±0.2 respectively), and since no antigen-driven T cell proliferation was observed between days 1 and 2 (Fig. S2), it is conceivable that the differences observed between dtg and stg mice may be explained by a defect in the recruitment of cells into the dLN. Indeed, when compared to dLNs from dtg mice, dLNs from stg mice that had received HA-loaded DCs showed not only higher numbers of CMTMR^+^ DCs, but also of CMTMR^−^, endogenous DCs (Fig. 2C). Furthermore, in the stg mice, the ratio of MHC class II high/low DCs in dLNs changed significantly as compared to non-draining LNs, indicating recruitment of immature or semi-mature DCs (Fig. 2D). This effect was suppressed in dtg mice, where the absolute numbers of endogenous DCs recovered from the dLN was significantly reduced compared to the stg mice. Similarly, the number of total CD8^+^ T cells at day 2 was significantly increased in the dLN of stg mice (Fig. 2E).

These results suggest that, in the presence of a high frequency of Tregs, recruitment of DCs and lymphocytes to the dLN is decreased during the initial phases of the immune response.

### Treg Frequency Determines the Chemokine Microenvironment of dLNs

The results reported above, together with the recent report by Rudensky and collaborators [21], prompted us to determine the chemokine levels in the dLN of stg and dtg mice upon antigen-specific immunization. To avoid biases due to the lower numbers of HA-pulsed DCs observed in the dLNs of dtg mice (Fig. 2), we decided to inject soluble peptide, which is able to travel via afferent lymphatics to the dLN and can be presented by resident DCs [27]. LPS was co-injected with the peptide in order to induce activation of DCs and achieve efficient T cell priming.

**Figure 3:**
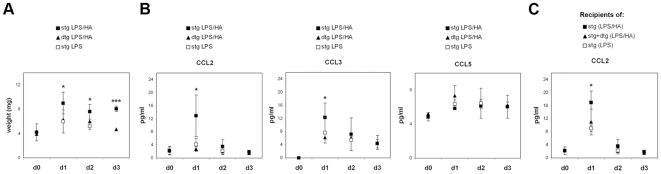
Effects of Tregs on chemokine production during an immune response. Stg and dtg mice were injected in the footpad with HA peptide (1 µg) together with LPS (0.5–1 µg). Control mice received LPS alone or nothing. Mice were sacrificed at different times after injection, as indicated. (A) dLNs were weighed and (B) CCL2, CCL3 and CCL5 chemokine content after homegenization was determined by ELISA. Shown are the pooled data from two independent experiments, with two to three mice per group in each experiment. (C) BALB/c mice were adoptively transferred with CFSE-labelled 6.5^+^ cells obtained from stg mice without or with equal numbers of 6.5^+^ cells isolated from dtg mice. The mice were immunized as described above. dLN were harvested and CCL2 amounts were determined by ELISA. Data from two pooled out of three independent experiments performed are shown.

Starting from one day after injection, the size and weight of the dLN were different in stg and dtg mice, with dLN from dtg mice being smaller, likely due to reduced arrival of immune cells (Fig. 3A). In agreement, we found that, at day 1, the proinflammatory chemokines CCL2 and CCL3, but not CCL5, were increased in the dLNs of stg mice, and that the values returned to basal levels already at day 3 (Fig. 3B). Importantly, in accordance with *in vitro* experiments recently reported [28], this chemokine secretion was the result of antigen-specific DC -T cell interactions, since LPS alone did not induce such an increase. In contrast, when a similar analysis was performed in dtg mice expressing higher frequency of Tregs, we found that the induction of both CCL2 and CCL3 after immunization was significantly suppressed, and this was not the case for CCL5 (Fig. 3B).

In order to eliminate the possibility that this difference in chemokine production was simply due to lower numbers of HA-specific Tconv cells in the dtg mice vs the stg mice, we performed adoptive transfers of CFSE-labelled 6.5^+^ cells from a stg mouse into BALB/c mice, in the absence or the presence of an equal number of 6.5^+^ cells isolated from dtg mice. Moreover, in this case, the ratio of Tconv∶Treg cells is higher than that in the experiments performed using dtg mice (2∶1 versus 1∶1). Adoptively transferred mice were immunized as described above, and chemokine production (Fig. 3C) and T cell activation in dLNs (Fig. S2) were analyzed. In accordance with the results obtained with stg and dtg mice, CCL2 production was significantly reduced when HA-specific T cells were primed in the presence of HA-specific Tregs. This was despite the fact that the percentage of CD69 positive cells among CFSE^+^ T cells and their early proliferative capacity were not significantly altered by the presence of regulatory T cells (Fig. S2), in agreement with previous data obtained using the same transgenic model [24]. Interestingly, endogenous DC migration to dLNs after 24 h of HA immunization was reduced in recipients of dtg cells as compared to recipients of stg ones (3.6+0.9×10^4^ vs 7.1+3×10^4^, respectively; p<0.05).

It is important to underline that, whilst in the experiments performed in the first part of our study (Fig. 2) we used antigen-bearing non-activated DCs, in the experiments described above (Fig. 3) we used LPS to activate endogenous DCs, thus suggesting that the effect of Tregs on chemokine microenvironment can be observed even in the presence of strong costimulatory signals.

Altogether, these results show that, during the early phases of an immune response, antigen-mediated DC-T cell interactions in LNs induce the production of pro-inflammatory chemokines that are required for further recruitment of immune cells. In LNs, Tregs counteract this priming-induced chemokine production and thus limit the very early phase of the immune response.

### Tregs Inhibit Antigen-Induced DC-T Cell Co-Localization within the LN

**Figure 4:**
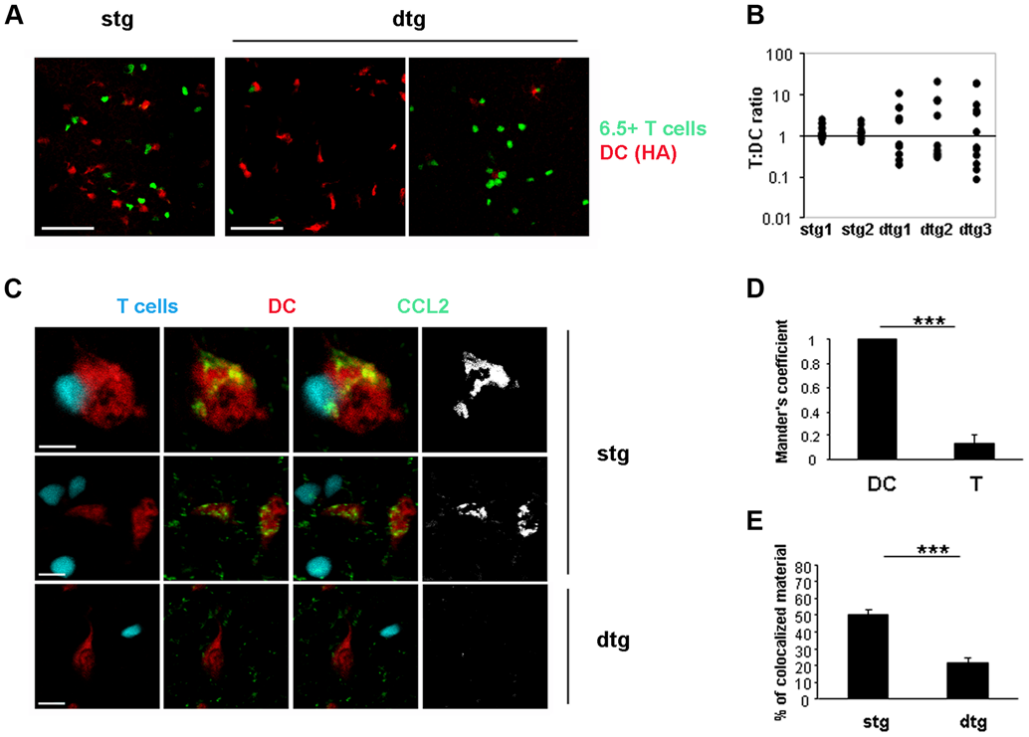
Effects of Tregs on antigen-specific DC-T cell attraction. CFSE-labelled 6.5^+^ T cells obtained from stg or dtg mice were adoptively transferred into BALB/c recipients. One day later, CMTMR-labelled, HA-loaded DCs were injected into the footpad. 24 h after DC transfer, mice were sacrificed and dLNs were frozen and cut for histological examination. (A) Representative images of two independent experiments are shown. Bar, 70 µm. (B) Quantitative analysis of the experiment in (A) on two LN sections from mice that had received T cells from stg donors (stg 1 and stg 2) and three LN sections from mice that had received T cells from dtg donors (dtg 1, dtg 2, dtg 3). Each dot represents the T cell/DC ratio (calculated as described in the Methods) for a single 250×250 µm quadrant. A non parametric ANOVA test was used and the difference between the higher 50% and the lower 50% values within each condition showed that there was no significant difference for the stg mice and a significant difference for the dtg mice (p<0.001). (C) Tissue sections were stained for CCL2. Bar, 10 µm. (D) Mander's co-localization coefficient was determined as described in the Methods and was used to understand if CCL2 had been produced by DCs or T cells, in mice that had received T cells from stg donors. (E) The “percentage of material co-localized” was determined as described in the Methods and represents the percentage of DC material (voxel signal intensity) that co-localizes with CCL2 in LN sections of mice that received T cells from stg or dtg donors.

It has been recently shown that CC chemokines like CCL2, CCL3 and CCL4, produced *in vitro* during cognate T cell-DC interactions, induce morphologic modifications and migration of DCs, both required for efficient T cell priming [28]. Furthermore, it was shown that CCL3 and CCL4 guide the recruitment of naive CD8^+^ T cells to sites of antigen-driven interactions between TLR-activated DCs and CD4^+^ T cells, optimizing memory CD8^+^ T cell responses [29]. In order to investigate whether the altered chemokine microenvironment observed in the presence of high Treg frequency could also affect the capacity of DCs to interact with T cells within the LN, we performed fluorescence microscopy analyses of dLN sections. CFSE-labelled 6.5^+^ T cells from stg or dtg mice were adoptively transferred into BALB/c mice and, one day later, CMTMR-labelled, HA-loaded DCs were injected in the footpad of the recipient mice. Mice were sacrificed one day after DC injection and the dLN were prepared for histology. CMTMR-positive DCs distributed throughout the T cell zones of the LN and many, but not all, were in the paracortex, as previously described using different experimental conditions [30]. In mice that had received T cells from the stg mice, confocal analysis of dLN sections showed that HA-specific T cells and HA-loaded DCs were in close proximity (Fig. 4A, left pannel). This co-localization was antigen-driven since it was not observed in the contra lateral dLN, where unloaded DCs were injected (Fig. S3). In contrast to what was observed in recipients of stg cells, in mice that received T cells from dtg mice, the majority of the HA-loaded DCs were not in close proximity with the HA-specific T cells (Fig. 4A, middle and right panels). In order to provide a quantitative estimate of this result, the T cell/DC ratio was calculated throughout the LN sections within 250×250 µm quadrants containing at least one red DC (Fig. 4B). This analysis demonstrated that in the dLN of mice that had received T cells from stg donors, the T cell/DC ratio was close to 1 in all quadrants, whereas in LNs enriched with Tregs the T/DC ratios were distributed over a range of values between 0 and 20. The means of the top two and bottom two quartiles of the values for the stg mice were not significantly different, whilst in dtg mice the difference was statistically significant (p<0.001, non-parametric ANOVA), suggesting that the dtg-derived population followed a bimodal distribution (Fig. 4B) with quadrants rich in T cells but poor in DCs and other quadrants rich in DCs but poor in T cells. This difference was not a reflection of an altered ratio of T cells and DCs within the entire LNs of recipient mice having received the dtg cells, because the total T cell/DC ratio, as calculated by flow cytometry on collagenase-digested dLNs, was not significantly different between the two types of recipients (data not shown).

Altogether, these results indicate that in the lymphoid microenvironment, Tregs inhibit antigen-driven chemokine production as well as co-localization of antigen-bearing DC and antigen-specific T cells.

In order to determine the source of CCL2 production as well as the main cellular target of Tregs during an antigen-specific immune response, we performed CCL2 staining of the histological sections shown above (Fig. 4C). CCL2 staining in the dLN that had received cells from a stg mouse was evident and mainly restricted to the cytoplasm of CMTMR-positive, antigen-loaded DCs. This was confirmed by the co-localization analysis expressed as Mander's coefficient (Fig. 4D), which was 0.997 for the CCL2-DCs pair (with 1.0 being the maximal co-localization) and 0.224 for CCL2-T cells (with 0 meaning no co-localization at all). Thus, in our experimental conditions, CCL2 was mainly produced and secreted by antigen-presenting DCs and not by activated T cells. In LNs that had received cells from dtg mice, CCL2 staining was restricted to antigen-pulsed DCs too, but, in agreement with our ELISA data, it was significantly reduced in intensity (Fig. 4E). A similar reduction was observed in the case of CCL3 when antigen-loaded DCs were analyzed in LNs that had received 6.5^+^T cells from stg or dtg mice (% of co-localized material: stg, 75.97±5.51; dtg: 48.07±3.54; p<0.01). In agreement with previous data [28], CCL3 staining was not restricted to DCs only but detectable also in T cells, although at lowers levels (Mander's coefficient for recipients of 6.5^+^ T cells from stg donors: DCs, 0.98±0.01; T cells 0.70±0.04; p<0.0001).

These results indicate that Tregs inhibit CCL2 and CCL3 production by antigen-presenting DC and thus limit recruitment of inflammatory cells into the LNs and T – DC co-localization within the LN.

## Discussion

Regulated migration of T cells and DCs from the periphery to and within the lymphoid tissues is a key element in the induction of immune responses [31]. *In vivo* imaging experiments have shown that lymphocytes entering the T-cell zones move randomly over densely packed networks of dendritic cells (DCs) and fibroblastic reticular cells (FRCs) [32], [33] . This motility is driven by CCR7-binding chemokines and may be pivotal for T cells to find their proper partners among numerous other cells. Besides CCL21, other chemokines produced in lymph nodes may coordinate specific encounters between cells. Thus, CCL3 and CCL4 seem to be involved in recruitment of naïve CD8^+^ T cells, which can upregulate CCR5 expression during inflammation, to sites where they can receive help from CD4^+^ T cells [34] .

Here we show that inflammatory chemokines, such as CCL2 and CCL3, are produced during the early phases of an antigen-specific immune response *in vivo*. Production of chemokines in the lymphoid tissues is important to recruit other immune cells and thus potentiate the response to antigens. Indeed, we found that the presence of antigen-presenting DCs in LNs induces recruitment of unloaded, immature or semimature DCs that substantially contribute to the reported increase in total LN cellularity [26]. In accordance, it has been shown that sustained T cell activation and proliferation require antigen presentation by migratory DCs [35] and that the entrance of blood-derived, inflammatory DCs into LNs depends on CCR2 [36]. We hypothesize that this antigen-induced, chemokine-driven recruitment of immune cells into the lymphoid tissue is one of the main targets of Treg action *in vivo*. We found that Tregs inhibit the early chemokine production occurring in LNs in response to antigen-specific DC-T cell interaction. Accordingly, dLNs enriched in Tregs were less efficient in recruiting inflammatory cells and in enhancing the immune response.

These results are in agreement with a previous elegant study showing that total ablation of polyclonal natural Tregs during viral infection resulted in an increase of certain proinflammatory chemokines within the dLNs, resulting in trapping of effector cells within the LN and poor viral clearance at the infection site [21]. In our study, we used a tunable antigen-specific approach that allowed us to demonstrate *in vivo* that: i) the production of pro-inflammatory chemokines is due to antigen-specific DC-T cell interactions; ii) changes in the Tconv∶Treg ratios have a strong impact on the lymphoid chemokine microenvironment; iii) chemokines released during antigen-driven interactions - at least CCL2 - are produced by antigen-presenting DCs; iv) Tregs block CCL2 production by antigen-presenting DCs, most likely inhibiting their ability to recruit other inflammatory cells and to co-localize with antigen-specific T cells within the LNs.

The fact that Tregs have a direct effect on the capacity of DCs to secrete chemokines is extremely interesting, especially when considering that, in our experimental systems, Tregs did not significantly affect the very early T cell response, as measured by up-regulation of the activation marker CD69. Although *in vitro* polyclonal Tregs can inhibit the production of CCR5 ligands by conventional T cells [37], our data indicate that, *in vivo*, the Treg target is mainly represented by DCs. This is in agreement with previous *in vivo* studies that have observed Treg-DC interactions within LNs, but could not report evidence for Treg-Tconv stable contacts [9], [10].

Although the precise mechanism by which Treg cells affect chemokine production induced by DC-T cell interactions remains to be determined, our data are in accordance with recent views on the mechanisms of regulatory T cells [8] indicating that DCs are an important target for regulation *in vivo*.

Altered Treg frequencies in blood, LNs and peripheral tissues have been reported in association with several pathological conditions (reviewed in [38], [39]). For example, cancer growth induces expansion of the Treg population through several mechanisms [40], [41], [42] and elevated Treg frequencies have been observed in patients affected by various types of malignancies (reviewed in [22]). On the other hand, several therapies are being tested to promote Treg expansion, development and survival *in vivo*, with the final aim of treating a variety of immunologic diseases ranging from autoimmunity to transplantation to allergy and asthma [43], [44]. Our data indicate that by increasing the Treg∶Tconv ratio it is indeed possible to switch off the inflammation associated to T cell antigen-recognition and thus provide further scientific support to the development of Treg-based therapies.

## Supporting Information

Figure S1 Undetectable apoptosis among HA-loaded DCs injected into stg and dtg mice. CMTMR-positive, HA-loaded DCs were injected into the footpad of stg or dtg mice. 48 h later, dLNs were recovered, frozen, fixed with PFA 4% and labelled with the polyclonal antibodies for Caspase 3 and Caspase 9 (1∶100, Cell Signaling Technology) as well as with the Tunel assay (according to the procedure suggested by the supplier, Roche). Magnification 60X. Bar, 5 µm. The inset shows a representative positive staining of a sporadic cell on the same section, although these did not correspond to CMTMR-positive cells. Magnification 60X with zoom. Bar, 5 µM
(2.00 MB TIF)

Figure S2 Poor co-localization of unloaded DCs and HA-specific T cells. Mice were adoptively transferred with CFSE-labelled 6.5+ cells and one day later were injected with CMTMR-positive DCs that were loaded with the HA peptide (shown in Figure 4) or not loaded, as control (shown here), in the contra-lateral footpad. 24 h later, dLNs were recovered, frozen and cut for histological examination. Shown is one representative dLN having received unloaded DCs; the left hand panel shows the internal region of the dLN while the right hand panel shows the cortical region of the LN. Magnification 60X. Bar, 40 µm
(0.19 MB TIF)

Figure S3 Poor co-localization of unloaded DCs and HA-specific T cells. Mice were adoptively transferred with CFSE-labelled 6.5 cells from stg mice and one day later were injected with CMTMR-positive DCs that were loaded with the HA peptide (shown in Figure 4) or not loaded, as control (shown here), in the contra-lateral footpad. 24 h later, dLNs were recovered, frozen and cut for histological examination. Shown is one representative dLN having received unloaded DCs; the left hand panel shows the internal region of the dLN while the right hand panel shows the cortical region of the LN. Magnification 60X. Bar, 40 µm
(1.23 MB TIF)
