# Supplementary figures and images for: Tunable Chemokine Production by Antigen Presenting Dendritic Cells in Response to Changes in Regulatory T Cell Frequency in Mouse Reactive Lymph Nodes
Source: PLoS One. 2009 Nov 6;4(11):e7696. doi: 10.1371/journal.pone.0007696 (PMC2770125; doi:10.1371/journal.pone.0007696)

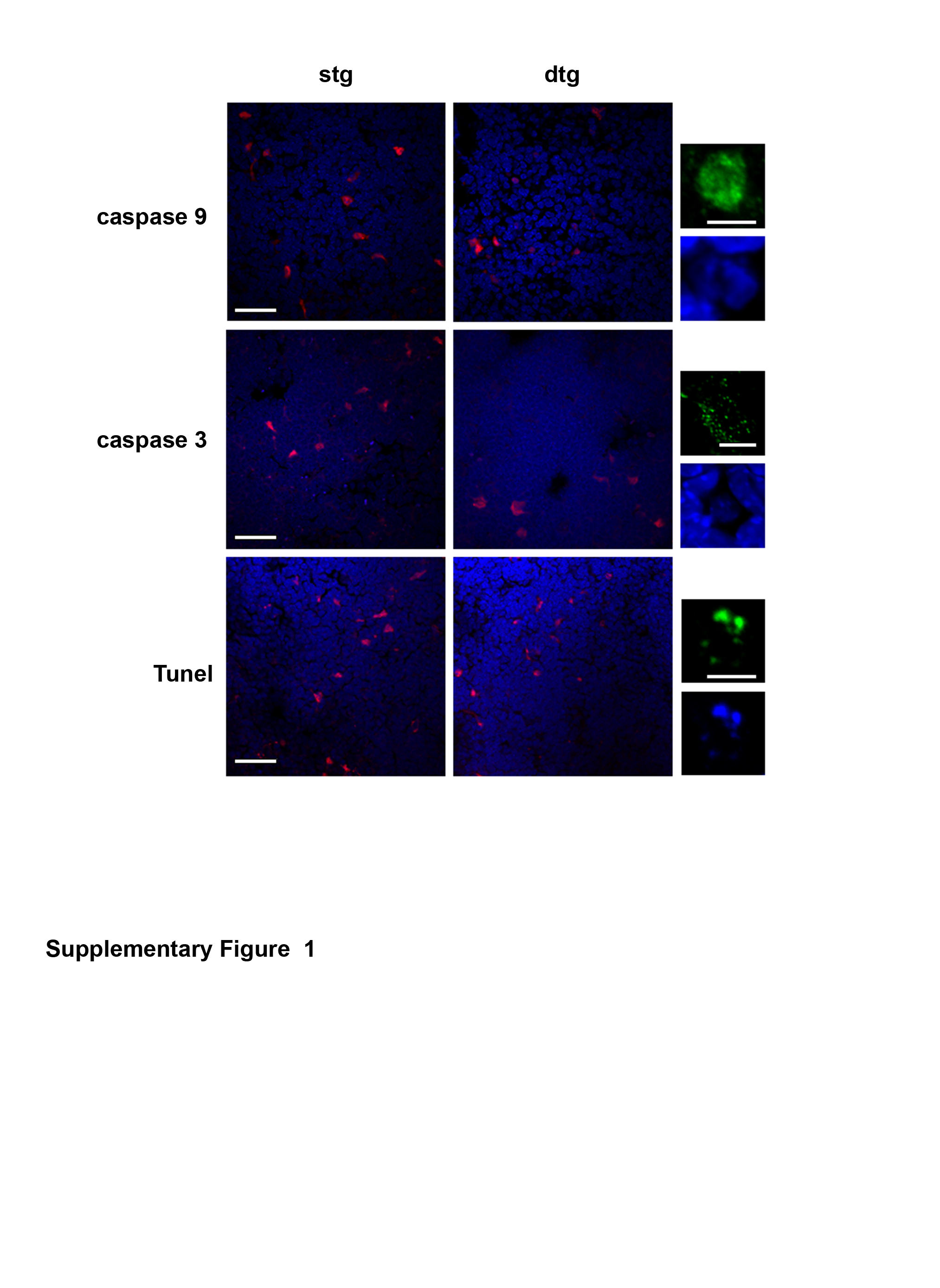

Supplement: Figure S1 — Undetectable apoptosis among HA-loaded DCs injected into stg and dtg mice. CMTMR-positive, HA-loaded DCs were injected into the footpad of stg or dtg mice. 48 h later, dLNs were recovered, frozen, fixed with PFA 4% and labelled with the polyclonal antibodies for Caspase 3 and Caspase 9 (1∶100, Cell Signaling Technology) as well as with the Tunel assay (according to the procedure suggested by the supplier, Roche). Magnification 60X. Bar, 5 µm. The inset shows a representative positive staining of a sporadic cell on the same section, although these did not correspond to CMTMR-positive cells. Magnification 60X with zoom. Bar, 5 µM (2.00 MB TIF) [file pone.0007696.s001.tif]

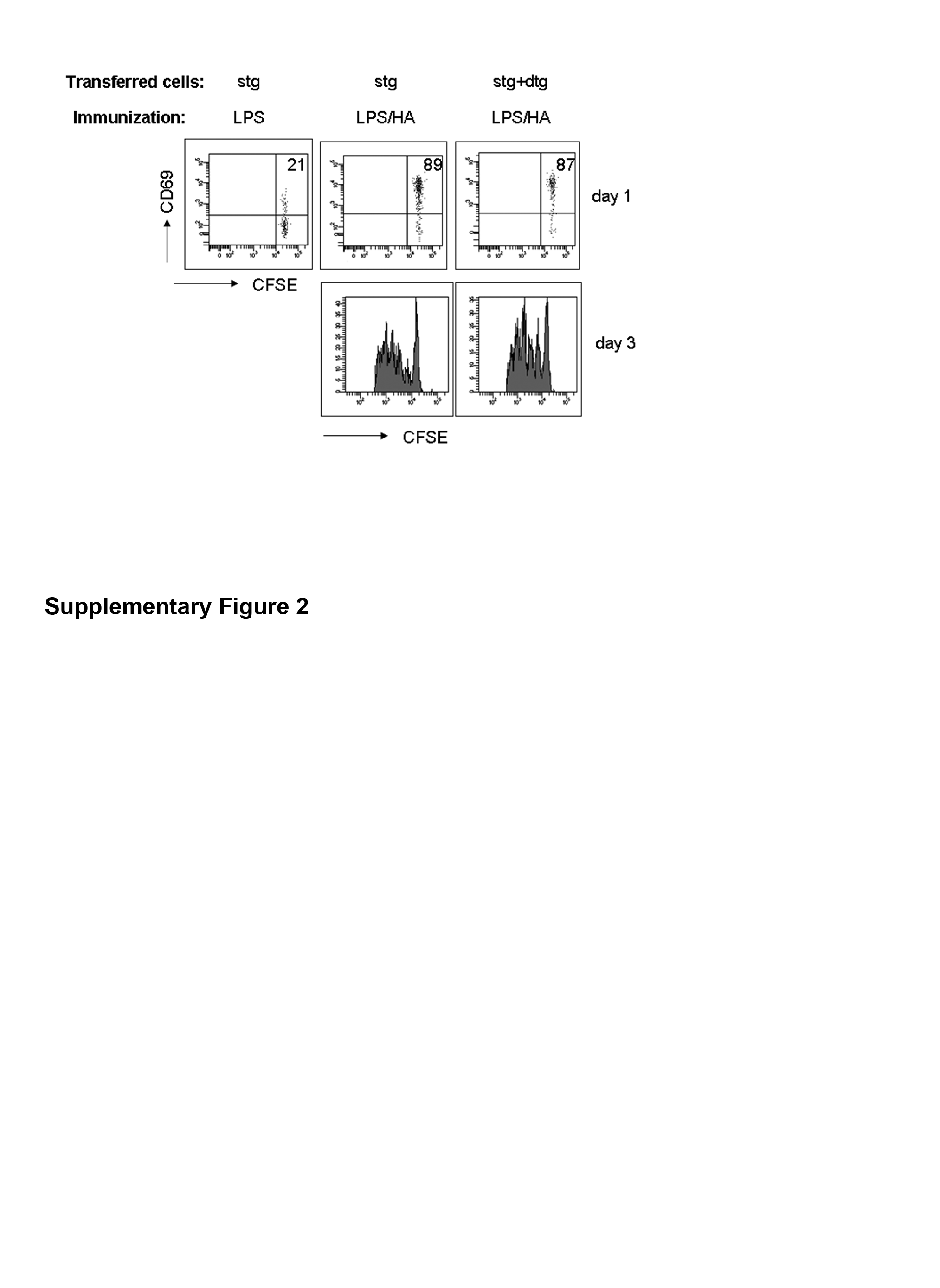

Supplement: Figure S2 — Poor co-localization of unloaded DCs and HA-specific T cells. Mice were adoptively transferred with CFSE-labelled 6.5+ cells and one day later were injected with CMTMR-positive DCs that were loaded with the HA peptide (shown in Figure 4) or not loaded, as control (shown here), in the contra-lateral footpad. 24 h later, dLNs were recovered, frozen and cut for histological examination. Shown is one representative dLN having received unloaded DCs; the left hand panel shows the internal region of the dLN while the right hand panel shows the cortical region of the LN. Magnification 60X. Bar, 40 µm (0.19 MB TIF) [file pone.0007696.s002.tif]

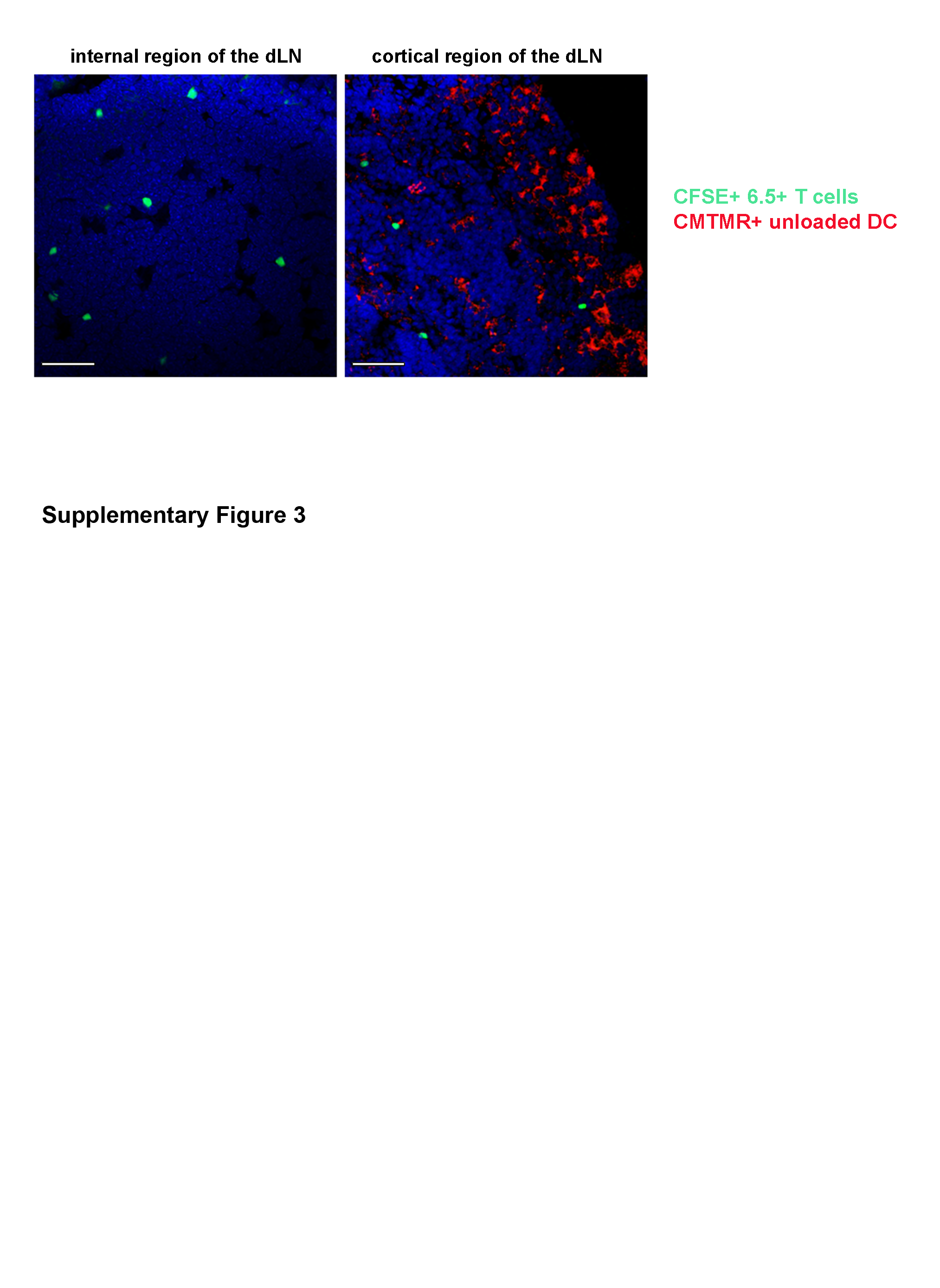

Supplement: Figure S3 — Poor co-localization of unloaded DCs and HA-specific T cells. Mice were adoptively transferred with CFSE-labelled 6.5 cells from stg mice and one day later were injected with CMTMR-positive DCs that were loaded with the HA peptide (shown in Figure 4) or not loaded, as control (shown here), in the contra-lateral footpad. 24 h later, dLNs were recovered, frozen and cut for histological examination. Shown is one representative dLN having received unloaded DCs; the left hand panel shows the internal region of the dLN while the right hand panel shows the cortical region of the LN. Magnification 60X. Bar, 40 µm (1.23 MB TIF) [file pone.0007696.s003.tif]
